# Therapeutic potential of digitoflavone on diabetic nephropathy: nuclear factor erythroid 2-related factor 2-dependent anti-oxidant and anti-inflammatory effect

**DOI:** 10.1038/srep12377

**Published:** 2015-07-24

**Authors:** Yang Yang, Gang Chen, Xiaolan Cheng, Zhiying Teng, Xueting Cai, Jie Yang, Xiaoyan Sun, Wuguang Lu, Xiaoning Wang, Yuanzhang Yao, Chunping Hu, Peng Cao

**Affiliations:** 1 Laboratory of Cellular and Molecular Biology, Jiangsu Province Academy of Traditional Chinese Medicine, Nanjing 210028, Jiangsu, China

## Abstract

Nuclear factor erythroid 2-related factor 2 (Nrf2) has emerged as a therapeutic target in many diseases, because it can induce antioxidant enzymes and other cytoprotective enzymes. Moreover, some Nrf2 activators have strong anti-inflammatory activities. Oxidative stress and inflammation are major components involved in the pathology of diabetic nephropathy. In the present study, we evaluated the Nrf2-dependent anti-oxidative and anti-inflammatory effects of digitoflavone in streptozotocin-induced diabetic nephropathy. The molecular mechanisms of digitoflavone were investigated *in vitro* using SV40-transformed mouse mesangial cells (SV40-Mes13). For the *in vivo* experiment, diabetes was induced in *Nrf2*+/+ and *Nrf2*−/− mice by STZ injection, and digitoflavone was administered 2 weeks after the STZ injection. Digitoflavone induced Nrf2 activation and decreased oxidative damage, inflammation, TGF-β1 expression, extracellular matrix protein expression, and mesangial cell hyperplasia in SV40-Mes13 cells. Digitoflavone-treated *Nrf2*+/+ mice, but not *Nrf2*−/− mice, showed attenuated common metabolic disorder symptoms, improved renal performance, minimized pathological alterations, and decreased oxidative damage, inflammatory gene expression, inflammatory cell infiltration, TGF-β1 expression, and extracellular matrix protein expression. Our results show that the anti-oxidative and anti-inflammatory effects of digitoflavone are mediated by Nrf2 activation and that digitoflavone can be used therapeutically to improve metabolic disorders and relieve renal damage induced by diabetes.

Mortality associated with diabetes doubled to 1.3 million deaths worldwide from 1990–2010^1^. Diabetic nephropathy is the major determinant of morbidity and mortality in patients with diabetes. Chronic hyperglycemia is a major initiator of diabetic nephropathy (DN)^2^; however, current therapies aimed at lowering blood glucose do not prevent the ultimate progression of DN, and co-treatment with renoprotective drugs often results in toxicity, limiting their efficacy. Therefore, the use of effective medications that specifically target DN represents a useful and needed addition to treatment strategies involving strict glycemic control.

Oxidative stress and inflammation induced by hyperglycemia and dyslipidemia play significant roles in the development of vascular complications in DN patients and animals^3^,^4^,^5^,^6^,^7^. Oxidative stress contributes to the onset and pathogenesis of diabetic nephropathy, and high glucose-induced renal damage is associated with excessive production of reactive oxygen species (ROS) under hyperglycemic conditions *in vitro*^2^,^8^. The development of DN is associated with significant inflammatory cell infiltration and increased plasma levels of C-reactive protein (CRP) and inflammatory cytokines such as vascular cell adhesion molecule-1 (VCAM-1) and IL-1^9^,^10^. Oxidative stress and inflammation are inseparably linked, as each begets and amplifies the other. Hence, therapies targeting oxidative stress and inflammation may effectively preserve normal renal function and prevent or slow the progression of DN.

The Nrf2-Keap1 (nuclear factor erythroid 2-related factor 2-Kelch-like ECH-associated protein 1) system is one of the most critical cytoprotective mechanisms protecting the body against oxidative stress, and it also plays an important role in reducing inflammation^11^,^12^,^13^,^14^. It regulates intracellular antioxidants, phase II detoxifying enzymes, and many other proteins that detoxify xenobiotics and neutralize ROS, and thus promotes cell survival and maintains cellular redox homeostasis^15^. Moreover, some Nrf2 activators also have strong anti-inflammatory activities^16^,^17^,^18^. A recent study found that Nrf2 is involved in the progression of renal disease^19^, and Nrf2 has been demonstrated to be an emerging therapeutic target for DN^20^,^21^. However, lessons gleaned from the phase II clinical trial of Nrf2 activator bardoxolone methyl suggest that new candidate Nrf2 activators and further mechanistic studies are needed before Nrf2 activators can be considered as a clinical treatment for DN.

Digitoflavone (3,0,4,5,7-tetrahydroxyflavone, Fig. 1A) is a member of the flavone subclass of flavonoids, which are enriched in vegetables and fruits^22^,^23^,^24^,^25^. Plants rich in digitoflavone have been used in Chinese traditional medicine to treat hypertension, inflammatory diseases, and cancer^26^. In recent studies, we found that digitoflavone produces several biological effects, including induction of cell cycle arrest, angiogenesis inhibition, nuclear factor kappa-light-chain-enhancer of activated B cells (NF-κB) down-regulation in apoptosis, and Nrf2 activation in chemoprevention^27^,^28^,^29^,^30^.

In the current study, we screened digitoflavone as an effective Nrf2 activator in mouse mesangial cells and explored its therapeutic potential to slow the progression of diabetic nephropathy using a streptozotocin (STZ)-induced model of diabetes.

## Materials and Methods

### Material

Digitoflavone, 2′, 7′-Dichlorofluorescin diacetate (DCFH-DA), Periodic (PAS) kit, Trichrom estain (Masson) kit, butylhydroquinone (T-BHQ) were obtained from Sigma-aldrich, USA. Digitoflavone was dissolved in DMSO for *in vitro* study. Maxima^®^ SYBR Green/ROX qPCR Master Mix (2×) and Maxima^®^ First Strand cDNA Synthesis Kit were purchased from Fermentas life science (Fermentas, MBI). RIPA lysis buffer, dihydroethidium (DHE) was purchased from Beyotime, China. Antibodies for immunoblot and immunohistochemical (IHC) analysis included anti-7, 8-dihydro-8-oxo-2’-deoxyguanosine (8-oxo-dG) (Millipore, USA); anti-Nrf2, glutamate-cysteine ligase catalytic subunit (GCLC), glutamate—cysteine ligase regulatory subunit (GCLM), heme oxygenase-1 (HO-1), β-actin, Fibronectin(FN), nitrotyrosine (Santa Cruz Biotechnology, CA, USA); anti-Collagen IV, TGF-β1 (Abcam, USA); anti-phosphorylation-p65 (p-p65), phosphorylation-nuclear factor of kappa light polypeptide gene enhancer in B-cells inhibitor, alpha (p-IκBα), phosphorylation-IκB kinase beta (IKKβ) (Cell Signaling Technology, MA, USA). Goat anti-rabbit IgG and goat anti-mouse IgG antibodies were purchased from LI-COR, Lincoln, NE, USA.

### Cell culture and MTT assay

The SV40-transformed mouse mesangial cells (SV40-Mes13) cells were purchased from Cell Bank of Shanghai Institute of Biochemistry and Cell Biology. Cells were normally cultured in DMEM containing 5% fetal bovine serum and 5.6 mM glucose, unless indicated otherwise. Cell viability was determined using the MTT assay.

### Transient transfection and analysis of luciferase reporter gene activity

The luciferase reporter assay was applied to investigate the transcriptional activity of Nrf2. The transient transfection and the ARE-luciferase reporter gene activity were carried out as previously described^30^. T-BHQ was set as a positive control.

### SDS –PAGE, Western blot analysis, Immunofluorescence staining and RT-PCR

Cultured cells or kidney tissues were lysed in sample buffer (Beyotime, China) and protein concentration was determined using Nanodrop 1000 Spectrophotometer (Thermo, USA). Protein samples were analyzed by Western blot as previously described^30^. Cells in logarithmic phase were seeded at the density of 60 ~ 70% confluence per well into 24-well chamber slides. After treatment with test samples for the indicated times, cells were analyzed by immunofluorescence staining as previously described^30^. Cultured SV40-Mes13 cells or kidney tissues were lysed in TRIZOL reagent (Invitrogen, Carlsbad, CA) and total RNA was isolated. Then RNA was analyzed by RT-PCR as previously described^30^. Primers used for the reactions were purchased from Genscript and the primer sequences are listed as followed: MCP-1(Forward: 5'-AGGTGTCCCAAAGAAGCTGTA-3', Reverse: 5'-ATGTCTGGACCCATTCCTTCT-3'), CSF-1(Forward: 5'-CCCATATTGCGACACCGAA-3', Reverse: 5'-AAGCAGTAACTGAGCAACGGG-3'), ICAM-1(Forward: 5'-GCCTTGGTAGAGGTGACTGAG-3', Reverse: 5'-GACCGGAGCTGAAAAGTTGTA-3'), VCAM-1(Forward: 5'-TGCCGAGCTAAATTACACATTG-3', Reverse: 5'-CCTTGTGGAGGGATGTACAGA-3').

### Cell proliferation assay

The rate of cell proliferation was measured two ways: 1) The iCELLigence system (Roche Applied Science, Germany and ACEA Biosciences, USA) allows real-time cell analysis (RTCA) via a system consisting of a microelectronic sensor array (MESA) 16× E-Plates coupled to a device station and an electronic sensor analyzer. The cell number, viability, morphology, and degree of adherence of cells in contact with the electrodes affects the local ionic environment and leads to an increase in the electrode impedance, which is presented as the cell index (CI). The SV40 MES13 cells (8,000 per well) were cultured in NG or HG medium with or without DIG, and cell growth was monitored for 30 h using the iCELLigence system. 2) Detection of Ki67 using indirect immunofluorescence.

### Animal experiments

All procedures involving animals were approved by the Institutional Animal Care and Use Committee of the Jiangsu Province Institute of Traditional Chinese Medicine and written up following the ARRIVE guidelines. Experiments were performed in accordance with published National Institutes of Health guidelines. *Nrf2*+/+ and *Nrf2*−/− C57BL/6 mice were purchased from the Jackson Lab and obtained through breeding of *Nrf2*+/−. All animals received water and food ad libitum. Eight-week-old mice received either sodium citrate (control) or STZ (50 mg/kg, pH 4.5, dissolved in sodium citrate) through intraperitoneal injection for 5 consecutive days. Two weeks following STZ injection, fasting glucose levels (4 h fast) were measured, and mice with a fasting glucose level above 250 mg/dL were considered diabetic and used for this study. Mice were randomly allocated into four groups (n = 8 per group) to receive treatment: 1) control = carboxymethyl cellulose (CMC), 2) STZ = CMC, 3) Digitoflavone (DIG) 1 = 25 mg/kg digitoflavone, 4) DIG 2 = 50 mg/kg digitoflavone. Digitoflavone at 25/50 mg/kg dose suspended in 0.5% CMC was given as gavage to mice and mice of control group and STZ group were given 0.2 mL 0.5% CMC solution every day from week 2 to week 12. Doses of digitoflavone were guided by published literature^30^ and tested in pilot studies to ensure Nrf2 activation within 24 h after gavage. Blood glucose level (4 h fast) and body weight were monitored throughout the course of treatment. At the end of 12 weeks of treatment, mice were put into clean metabolic cages one day before sacrifice, and urine was collected for 24 h. At week 12, mice were sacrificed and the kidney, blood, and urine were isolated for analysis.

### Measurements of creatinine, BUN, blood insulin and urinary albumin

Blood glucose level (4 h fast) was determined by glucometer (Ascensia Elite, Bayer, Leverkusen, Germany). Serum creatinine and usea nitrogen clearance were measured as an index of GFR. Urinary and blood creatinine, BUN, blood insulin and urinary albumin were detected by standard diagnostic kits (Jiancheng Biotech Co., Ltd., Nanjing, China) according to the manufacturer’s instructions.

### Renal morphology assessment and IHC analysis

Tissue sections from paraffin-embedded kidney were stained with HE, PAS, and Masson’s trichrome. For PAS-stained tissue sections, a five-grade method was used to evaluate the sclerosis in glomeruli as described^31^. For IHC analysis, the deparaffinized sections were boiled in sodium citrate buffer and primary antibody was used in a dilution of 1:100 for 4 °C overnight. The immunostaining was visualized using diaminobenzidine tetrahydrochloride (DAB), and the slides were counterstained with hematoxylin.

### Oxidative stress test

Oxidative DNA damage in the glomerulus was measured by the amount of 8-oxo-dG, deparaffinized kidney sections were treated with proteinase K (10 mg/mL ), RNase A (100 mg/mL), and 2 N HCl then stained by IHC with an anti-8-oxo-dG antibody. Oxidative protein damage was measured by the IHC analysis of nitrotyrosine.

Cellular ROS contents were measured by incubating SV40-Mes13 cells with 10 μM DCFH-DA for 30 min, and the fluorescence intensity was measured by flow cytometry. O_2_·^−^ level was measured by flow cytometry using 10 μM DHE for 30 min to obtained sufficient fluorescence signal.

### Statistical analysis

Results are expressed as mean ± SD. Statistical tests were performed using SPSS 15.0. Unpaired Student t tests were used to compare the means of two groups. For multiple comparisons between groups, a one-way ANOVA was performed to detect statistical differences. Differences within the ANOVA were determined using a Tukey’s post-hoc test. P value of less than 0.05 was considered to be statistically significant.

## Results

### Digitoflavone activates the Nrf2 pathway and diminishes mesangial ROS generation under hyperglycemic conditions

**Figure 1:**
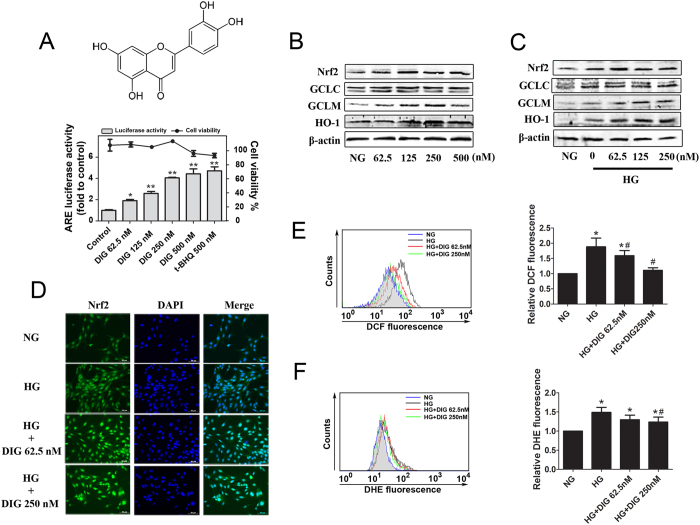
Digitoflavone activated Nrf2 diminishes mesangial ROS generation under hyperglycemic conditions. (**A**) Effects of digitoflavone on ARE-driven luciferase reporter activity in SV40-Mes13 cells. SV40-Mes13 cells were treated with various concentrations of digitoflavone for 24 h. Luciferase activity and cell viability were assayed in parallel as described in the materials and methods section. T-BHQ treatment represented an internal positive control. Top: chemical structure of digitoflavone. (**B)** and (**C**) Protein expression of Nrf2 and downstream targets was analyzed in SV40-Mes13 cells incubated in NG with various concentration of digitoflavone (**B**), HG, or HG + digitoflavone (**C**) in DMEM media for 24 h. (**D**) Nrf2 localization was assessed in SV40-Mes13 cells incubated in NG, HG, or HG + digitoflavone DMEM media for 24 h. (**E**) and (**F**) Total ROS levels (**E**) and O_2_·^−^ levels (**F**) in SV40-Mes13 cells incubated in NG, HG, or HG + digitoflavone DMEM media are reported. All data represent the mean ± SD of triplicate independent experiments. *P < 0.05 vs. NG group. **P < 0.01 vs. control mice. ^#^P < 0.05 vs. HG group.

Mesangial cells play a crucial role in dictating the function of glomeruli. SV40-Mes13 cells were used to study the molecular mechanism by which digitoflavone preserves renal function during the progression of diabetic nephropathy. Treatment of SV40-Mes13 cells with digitoflavone for 24 h increased antioxidant-response element (ARE)-luciferase activity (Fig. 1A, bottom). Parallel cell viability assays revealed no obvious cytotoxic effects (62.5 to 250 nM) (Fig. 1A, bottom). Western blot analysis demonstrated that digitoflavone significantly and dose-dependently increased protein abundance of Nrf2 and its downstream anti-oxidant targets (Fig. 1B).

To mimic hyperglycemic conditions in diabetic nephropathy, SV40-Mes13 grown in normal glucose (NG) (5.5 mmol/L) medium were shifted to either NG medium with 19.5 mmol/L mannitol or high glucose (HG) (25 mmol/L) medium in the presence or absence of digitoflavone. Increased Nrf2 expression and predominant nuclear localization of Nrf2 were observed in response to digitoflavone treatment (Fig. 1C,D). Similarly, protein levels of Nrf2 downstream targets—GCLM, GCLC and HO-1 were increased in response to HG and further enhanced by treatment with digitoflavone (Fig. 1C). Hyperglycemic conditions also activated the Nrf2 pathway (Fig. 1C,D) in cultured SV40-Mes13 cells, presumably through oxidative stress.

SV40-Mes13 cells cultured in HG media showed higher levels of ROS and the free radical superoxide anion (O_2_·^−^) in comparison with those cultured in NG media, and activation of Nrf2 by digitoflavone reduced ROS (Fig. 1E) and O_2_^−^ (Fig. 1F) levels.

### High glucose-mediated mesangial cell hyperplasia and inflammation can be antagonized by digitoflavone

**Figure 2:**
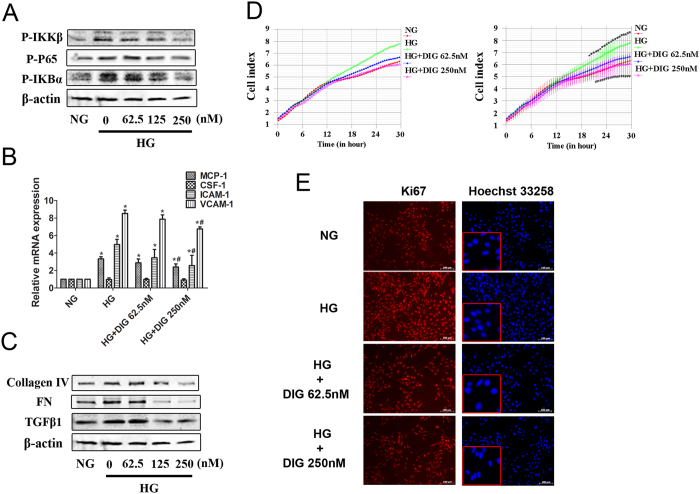
High glucose-mediated mesangial cell hyperplasia and inflammation can be antagonized by digitoflavone induced Nrf2. (**A**) Expression of NF-κB pathway proteins was assessed by immunoblot analysis in SV40-Mes13 cells incubated in NG, HG, or HG + digitoflavone DMEM media for 24 h. (**B**) Expression of NF-κB downstream genes was assessed by real-time PCR analysis in SV40-Mes13 cells. (**C**) Expression of TGF-β1 and downstream proteins was assessed by immunoblot analysis in SV40-Mes13 cells. (**D**) Cell growth of SV40-Mes13 cells incubated in NG, HG, or HG + digitoflavone DMEM media were monitored in real-time for 30 h (upper panel = average; lower panel = average with error). (**E**) Cell hyperplasia and death were assessed by Ki67 immunolabeling or Hoechst 33258 staining. All data represent the mean ± SD of triplicate independent experiments. *P < 0.05 vs. NG group. ^#^P < 0.05 vs. HG group.

High glucose induced phosphorylation of NF-κB, its upstream kinase IκB kinase beta (IKKβ), and NF-κB inhibitor nuclear factor of kappa light polypeptide gene enhancer in B-cells inhibitor alpha (IκBα); however, digitoflavone treatment reduced phosphorylation of NF-κB pathway members in a dose-dependent manner (Fig. 2A). High glucose also increased the expression of NF-κB downstream inflammatory cytokines MCP-1, as well as ICAM-1 and VCAM-1, and these effects were reversed by digitoflavone treatment (Fig. 2B).

Expression levels of TGF-β1 and its downstream effectors were analyzed under hyperglycemic conditions. Hyperglycemia up-regulated TGF-β1, FN and collagen IV in SV40-Mes13 cells, and these effects were suppressed by treatment with digitoflavone (Fig. 2C).

The SV40-Mes13 cells grown in HG media proliferated more over the course of the experiment than those grown in NG or in HG supplemented with digitoflavone (Fig. 2D). Immunofluorescence analysis of Ki67 showed that hyperglycemia caused mesangial cell hyperplasia, which was counteracted by digitoflavone-induced Nrf2 activation. No cell death was observed using Hoechst 33258 staining under any condition (Fig. 2E).

### Activation of Nrf2 by digitoflavone improves metabolic disorder indices in an STZ-induced diabetic model

**Figure 3:**
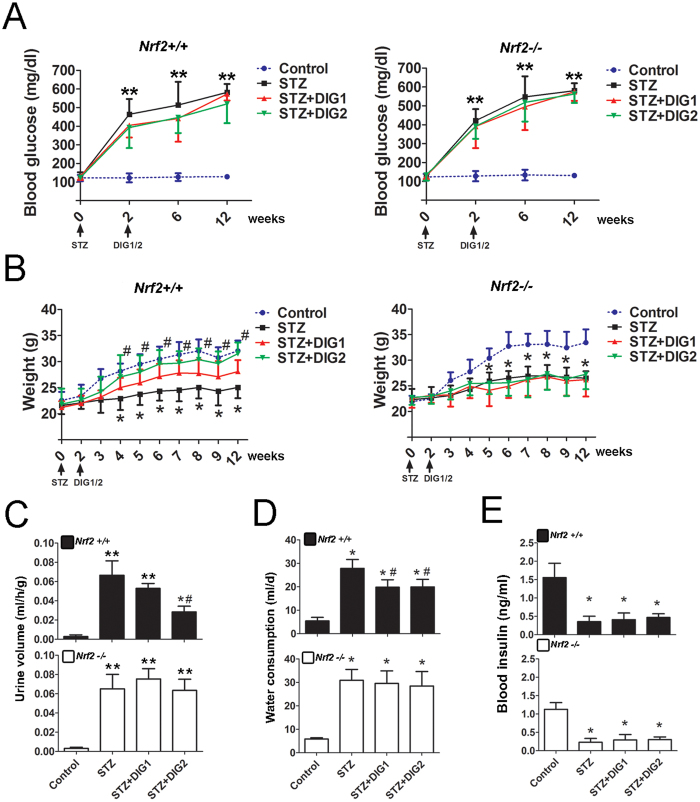
Activation of Nrf2 by digitoflavone improves metabolic disorder in a STZ-induced diabetic model. (**A**) The average level of blood glucose (4h fast) in each animal group is plotted. (**B**) Body weights of *Nrf2+/+* (left panel) and *Nrf2−/−* (right panel) animals are shown. (**C**) Urine output from *Nrf2+/+* (top panel) and *Nrf2−/−* (bottom panel) animals is shown. (**D**) Water consumption in the last 24 h before the mice were sacrificed is reported. (**E**) Blood insulin content is shown. Data are expressed as mean ± SD (n = 5). *P < 0.05 vs. control mice. **P < 0.01 vs. control mice. #P < 0.05 vs. STZ mice.

*Nrf2* knockout mice (*Nrf2−/−*) were used to study the effectiveness of dietary Nrf2 activator digitoflavone in diabetic nephropathy. STZ was used to induce diabetes in *Nrf2* wild-type (*Nrf2*+/+) and *Nrf2−/−* mice, and diabetes-related common metabolic disorder indices were measured. *Nrf2+/+* and *Nrf2−/−* mice treated with STZ showed significantly increased blood glucose levels (4 h fast) (Fig. 3A), urine production (Fig. 3C), and water uptake (Fig. 3D), and decreased weight gain (Fig. 3B). Importantly, only *Nrf2+/+* animals showed significantly alleviated metabolic dysfunction indices following digitoflavone treatment, demonstrating that this effect of digitoflavone was Nrf2-dependent. In addition, STZ significantly decreased insulin levels, and digitoflavone treatment did not alter insulin levels regardless of genotype (Fig. 3E), indicating that the protective effect of digitoflavone was insulin-independent.

### Digitoflavone alleviates renal damage induced in the STZ diabetic model

**Figure 4:**
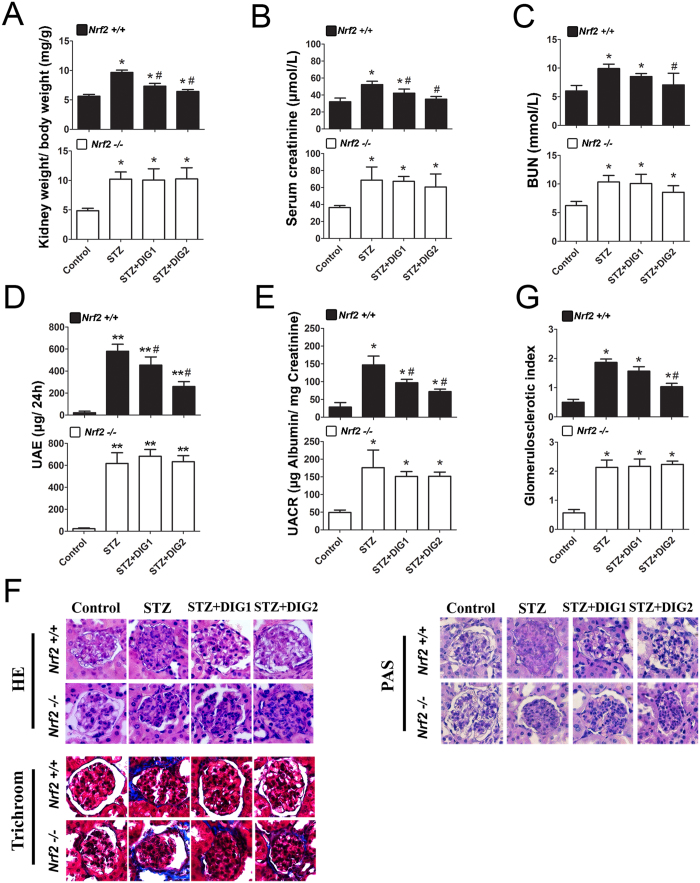
Digitoflavone alleviates renal damage induced in the STZ-diabetic model. (**A**) Kidney-to-body weight ratios are provided. (**B**) and (**C**) Serum creatinine and BUN content is reported. (**D**) and (**E**) Albuminuria was assessed by measuring UAE (**D**) and UACR (**E**). Kidney tissue sections from each mouse were subjected to HE, PAS and trichrome staining. (**F**) A representative image from 1 mouse per group is shown. G: PAS-stained tissues were used for semiquantitative scoring to obtain the glomerulosclerotic index. Data are expressed as mean ± SD (n = 5). *P < 0.05 vs. control mice. **P < 0.01 vs. control mice. ^#^P < 0.05 vs. STZ mice.

Functional and pathological changes in the kidney were measured to investigate the therapeutic effects of digitoflavone on kidney function in the STZ diabetes model. The ratio of kidney weight to body weight in all STZ-injected groups was higher than that of the control group, and this ratio was lowered significantly by treatment with digitoflavone in *Nrf2+/+* mice (Fig. 4A). Diabetic mice exhibited higher serum creatinine and BUN, but chronic treatment with digitoflavone (50 and 100 mg/kg/day) significantly and dose-dependently reduced serum creatinine (Fig. 4B) and blood urea nitrogen (Fig. 4C). UAE and UACR were tested at week 12. STZ increased UAE and UACR in all treated groups; however, this increase was reversed by digitoflavone treatment in *Nrf2+/+* mice only (Fig. 4D,E).

In agreement with the urine and blood analysis, histological examination showed that digitoflavone treatment suppressed STZ-induced pathological changes in the glomerulus. Glomerular lesions were observed in HE-stained tissue sections from STZ-injected mice (Fig. 4F, HE panel). Treatment with digitoflavone effectively restored the normal morphology of glomeruli in *Nrf2+/+*, but not *Nrf2−/−*, mice (Fig. 4F, HE panel). PAS staining showed glomerulosclerosis in the STZ-treated group, which was significantly ameliorated in the digitoflavone-treated *Nrf2+/+*, but not *Nrf2−/−*, mice (Fig. 4F, PAS panel, and Fig. 2G). STZ treatment resulted in collagen deposition inside the glomeruli and in the periglomerular area, which was reduced in digitoflavone-treated *Nrf2+/+*, but not *Nrf2−/−*, mice (Fig. 4F, trichrome panel).

### Digitoflavone-induced activation of the Nrf2 pathway confers protection against renal oxidative damage

**Figure 5:**
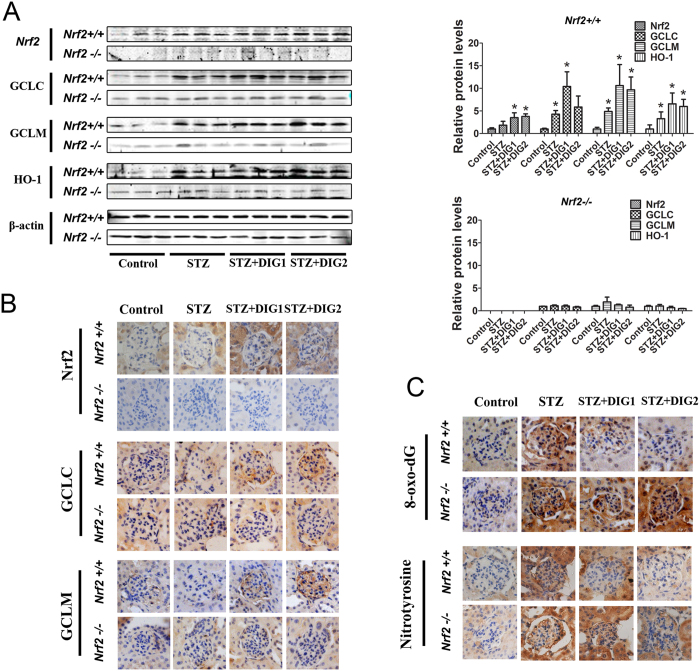
Digitoflavone-induced activation of Nrf2 pathway confers protection against renal oxidative damage. (**A**) Whole kidney lysates from 3 mice per group were subjected to immunoblot analysis with antibodies against Nrf2, GCLC, GCLM, HO-1, and β-actin. The intensity of bands from replicate immunoblots was quantified and plotted (right panel, bar graphs). (**B**) Fixed kidney tissues were stained with antibodies against Nrf2, GCLC, and GCLM. (**C**) 8-oxo-dG and nitrotyrosine staining assessed oxidative damage in the kidney, and representative images are provided. Data are expressed as mean ± SD (n = 3). *P < 0.05 vs. control mice. ^#^P < 0.05 vs. STZ mice.

To demonstrate that the beneficial effect of digitoflavone against renal damage was Nrf2-dependent, expression levels of Nrf2 and its downstream targets, as well as oxidative damage in the kidney, were assessed. Treatment with digitoflavone markedly increased protein levels of Nrf2, GCLC, GCLM, and HO-1 (Fig. 5A,B). Importantly, digitoflavone treatment significantly reduced oxidative damage in the kidneys of *Nrf2+/+* mice, as measured by the local formation of 8-oxo-dG and nitrotyrosine in glomerular tissue (Fig. 5C,D). Treatment with digitoflavone did not reduce oxidative damage or increase expression of GCLC, GCLM, or HO-1 in *Nrf2−/−* mice.

### Digitoflavone reduced interstitial inflammatory cell infiltration and inflammatory gene expression through an Nrf2-dependent mechanism in the diabetic kidney

**Figure 6:**
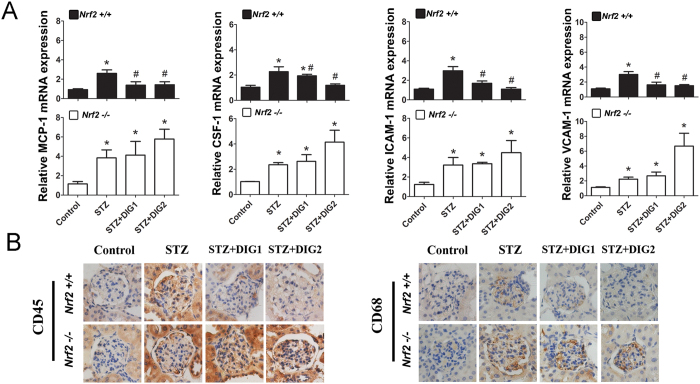
Digitoflavone reduces renal inflammatory gene expression and inflammatory cell infiltration. (**A**) Whole kidney RNA from 3 mice per group was subjected to RT-PCR analysis of MCP-1, CSF-1, ICAM-1, and VCAM-1 gene expression. (**B**) Renal inflammatory cell infiltration was analyzed by IHC. Data are expressed as mean ± SD (n = 3). *P < 0.05 vs. control mice. ^#^P < 0.05 vs. STZ mice.

The development of DN is associated with significantly increased inflammatory cytokine expression and inflammatory cell infiltration. Real-time PCR (RT-PCR) revealed that STZ-induced diabetes was associated with significantly up-regulated MCP-1 (*Mcp1*), CSF-1 (*Csf1*), ICAM-1 (*Icam1*), and VCAM-1 (*Vcam1*) expression in *Nrf2+/+* and *Nrf2−/−* mice, and these changes in expression were reversed by digitoflavone in *Nrf2+/+* mice only (Fig. 6A). Immunohistochemical staining for CD45 and CD68 was performed in kidney tissue samples from digitoflavone-treated or untreated diabetic mice to observe focal interstitial inflammatory cell infiltration. Diabetic mice showed significantly increased infiltration of CD45-positive (CD45+) leukocytes and CD68-positive (CD68+) macrophages in interstitial areas, while the control kidneys showed fewer leukocytes and no macrophages. Digitoflavone dose-dependently reduced renal CD45+ leukocyte and CD68+ macrophage infiltration in *Nrf2+/+* animals only (Fig. 6B).

### Nrf2 activation regulates TGF-β1 and extracellular matrix (ECM) deposition

**Figure 7:**
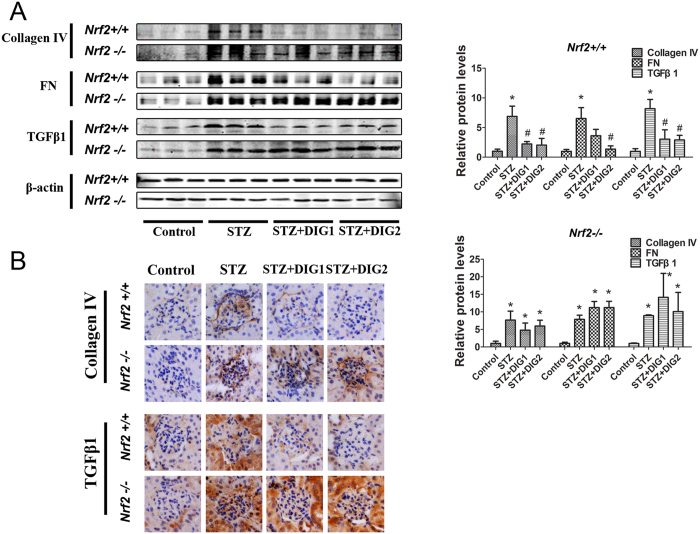
Digitoflavone activated Nrf2 reduces TGF-β1 expression and ECM deposition. (**A**) Immunoblot analysis was performed on lysates from the whole kidney (left panel), and band intensities were quantified (right panel, bar graphs) and reported as relative expression to control animals. (**B**) Fixed kidney tissue was analyzed by IHC. Data are expressed as mean ± SD (n = 3). *P < 0.05 vs. control mice. ^#^P < 0.05 vs. STZ mice.

A previous study found a negative association between Nrf2 expression and TGF-β1 expression^21^. Corroborating these previously published results, basal expression of TGF-β1 in *Nrf2−/−* mice was higher than that of *Nrf2+/+* mice (Fig. 7A,B). Diabetes induced by STZ was associated with significantly up-regulated protein levels of TGF-β1 and its downstream effectors FN and collagen IV, and these effects were significantly diminished by treatment with digitoflavone in the *Nrf2+/+* animals only (Fig. 7A,B).

## Discussion

Oxidative stress and inflammation are critical mediators of the pathogenesis and progression of chronic kidney disease (CKD), acting in a self-perpetuating cycle in which oxidative stress causes inflammation by several mechanisms, including the activation of NF-κB. In turn, inflammation causes oxidative stress via production of reactive oxygen, nitrogen, and halogen species by activated leukocytes and resident cells^32^. Our previous studies showed that digitoflavone served as an NF-κB inhibitor and Nrf2 activator in different disease models^29^,^30^. In the current study, the anti-oxidative and anti-inflammatory effect of digitoflavone in diabetic nephropathy was clearly demonstrated both *in vitro* and *in vivo*.

Constitutive Nrf2 activity is crucial in maintaining redox balance under normal conditions, and its induction in response to oxidative stress, with consequent transcription of cytoprotective genes, represents an important defense system against oxidative stress^32^. Digitoflavone-induced Nrf2 activation in cultured mouse mesangial cells has been confirmed under NG and HG conditions, and its downstream antioxidant proteins GCLC, GCLM, HO-1, which comprise the main antioxidant effectors of digitoflavone-induced Nrf2 activation, were also up-regulated^30^. Several reactive oxygen intermediates can be generated in the progression of diabetic nephropathy: O_2_·^−^, the non-radical hydrogen peroxide (H_2_O_2_), the highly reactive hydroxyl free radical (·OH), peroxynitrite (ONOO^−^), and singlet oxygen (^1^O_2_). Among the ROS, attention has mainly been focused on superoxide anion production^33^. Reduced superoxide anion and total ROS levels were found in the digitoflavone-treated mouse mesangial cells under HG conditions.

The digitoflavone-induced Nrf2-dependent antioxidant response is accompanied by a reduced inflammatory response, which was associated with decreased phosphorylation of members of the NF-κB signaling pathway (IKKβ, IκBα, and NF-κB) and down-regulation of its downstream inflammatory cytokines MCP-1, ICAM-1, and VCAM-1. Several mechanisms have been demonstrated to explain the relationship between Nrf2 signaling and NF-κB signaling, including Keap1, which may bind to IKKβ and thus inhibit NF-κB signaling^34^, and HO-1, which may limit NF-kB activity by inhibiting IκBα degradation^35^. However, because IKKβ ETGE motifs do not exist in rodent cells^34^, Keap1 cannot negatively regulate IKKβ in mouse mesangial cells. Because of this limitation of the action of Keap1 in rodents, the anti-inflammatory effect of digitoflavone may be caused by its anti-oxidant activity in mouse mesangial cells. Further studies are merited to determine whether regulation of the Nrf2/Keap1 system by digitoflavone can inhibit IKKβ in human cells. We also showed that digitoflavone-induced Nrf2 activation can reduce cell hyperplasia and down-regulate expression of TGF-β1 and its downstream effectors in mouse mesangial cells under HG conditions.

In addition to demonstrating the therapeutic potential of digitoflavone in suppressing the pathogenesis of diabetic nephropathy, we also sought to determine the mechanism by which digitoflavone-induced Nrf2 activation produces beneficial effects. *Nrf2* wild type and knockout mice were subjected to the STZ-induced diabetic model in an *in vivo* study. The observed mild reduction in blood glucose might have contributed to the effects of digitoflavone in *Nrf2+/+* mice. We also found that digitoflavone had no effect on insulin levels, and the reduction in blood glucose may have been insulin-independent. The altered gluconeogenesis and glycolysis observed in the *Nrf2*^*−/−*^ mice^36^ suggests that the blood glucose reduction in the DIG-treated *Nrf2*^*+/+*^ mice may be partially attributable to the Nrf2-mediated glucose metabolism and glycogen formation in the liver. Alternatively, DIG might improve hepatic insulin sensitivity by suppressing the expression of the sterol regulatory element-binding protein (SREBP)-1^37^. The published literature in this field supports the notion that Nrf2 activators influence glucose up-take in fibroblasts^38^ and play a central role in the acquisition of insulin resistance induced by oxidative stress in cardiomyocytes^39^.

The Nrf2-dependent antioxidant response might be the main contributing factor to the protective effect of digitoflavone in diabetic nephropathy. The role of oxidative stress in the pathogenesis of diabetic nephropathy has gained increasing research attention in recent years^40^,^41^. In the current study, digitoflavone treatment significantly increased expression of Nrf2 and its downstream anti-oxidant proteins, and this effect was accompanied by reduced oxidative damage in the glomerular tissues of STZ-treated *Nrf2*+/+ mice. These results indicate the importance of the Nrf2-dependent antioxidant response in ROS neutralization and alleviation of oxidative damage in the kidney.

We also showed that digitoflavone-induced Nrf2 activation decreased inflammation in mice with STZ-induced diabetic nephropathy. Inflammation is crucial in promoting the development and progression of DN^42^. Leukocytes produce pro-inflammatory cytokines that can induce resident renal cells to produce a spectrum of chemokines, including inflammatory cytokines MCP-1 and CSF-1 and adhesion molecules ICAM-1 and VCAM-1^42^. In accordance with the *in vitro* study, digitoflavone treatment significantly alleviated STZ-induced changes in MCP-1, CSF-1, ICAM-1, and VCAM-1 gene expression in *Nrf2*+/+ mice only. In human DN, leukocytes and macrophages accumulate in the glomeruli and interstitium, even in the early stages of the disease^43^. In the STZ-induced diabetic nephropathy model, both *Nrf2*+/+ and *Nrf2−/−* mice showed accumulation of leukocytes and macrophages in the kidney, and digitoflavone treatment significantly reduced inflammatory cell infiltration in *Nrf2*+/+ mice. Taken together, our *in vitro* and *in vivo* results show that digitoflavone induced Nrf2-dependent anti-oxidative effects that modulated inflammation in STZ-induced diabetic nephropathy, leading to down-regulation of inflammatory genes and reduced inflammatory cell infiltration.

Digitoflavone-mediated protection in diabetic nephropathy may also be mediated by the negative regulatory effect of Nrf2 activation on TGF-β1, a major profibrotic mediator of diabetic nephropathy. Subsequently, we found that digitoflavone treatment reduced expression of TGF-β1 and its downstream ECM proteins in mice with STZ-induced diabetic nephropathy and mouse mesangial cells.

Our findings indicate that the therapeutic benefit of digitoflavone in diabetic nephropathy is Nrf2-dependent. In addition to its antioxidant function, Nrf2 also negatively regulates inflammation and expression of TGF-β1 and ECM proteins. Our results provide convincing experimental evidence that dietary digitoflavone activates Nrf2 and can be used therapeutically to improve metabolic disorder and relieve kidney damage induced by diabetes. This study lays the foundation for clinical evaluation and the ultimate development of Nrf2 activator digitoflavone as a clinically used agent to prevent the onset and progression of diabetic nephropathy.

## Additional Information

**How to cite this article**: Yang, Y. *et al.* Therapeutic potential of digitoflavone on diabetic nephropathy: nuclear factor erythroid 2-related factor 2-dependent anti-oxidant and anti-inflammatory effect. *Sci. Rep.* **5**, 12377; doi: 10.1038/srep12377 (2015).
